# Serum Kisspeptin and AMH Levels Are Good References for Precocious Puberty Progression

**DOI:** 10.1155/2020/3126309

**Published:** 2020-11-20

**Authors:** Jiang Xue, Wei Song, Min Si, Chao Sun, Kailin Li, Wei Wang, Shuang Liang, Yanfeng Xiao

**Affiliations:** ^1^Department of Pediatrics, The Second Affiliated Hospital of Xi'an Jiaotong University, Xi'an, Shaanxi, China; ^2^Department of Pediatrics, The Second Hospital, Cheeloo College of Medicine, Shandong University, Jinan, China; ^3^Department of Intensive Care Unit, Jinan Central Hospital Affiliated to Shandong First Medical University, Jinan, China; ^4^Department of Central Laboratory, The Second Hospital, Cheeloo College of Medicine, Shandong University, Jinan, China; ^5^Department of Interventional Medicine, The Second Hospital, Cheeloo College of Medicine, Shandong University, Jinan, China

## Abstract

**Aim:**

The aim of this study was to evaluate the levels of kisspeptin and AMH in children with PT or CPP or controls to provide a reference for diagnosis and prognosis.

**Methods:**

38 Chinese children with central precocious puberty (CPP), 38 Chinese children with premature thelarche (PT), and 75 controls were recruited.

**Results:**

In CPP girls, AMH levels decreased significantly compared to control girls at T2 stage. Compared with the PT and control groups, AMH is the lowest in girls in the CPP group at T3 stage. Kisspeptin decreased significantly in girls in the PT group and increased significantly in girls in the control group from T2 stage to T3 stage. At T3 stage, kisspeptin was significantly higher in girls in the CPP and control groups than in the PT group. In the control group, kisspeptin was significantly higher in boys than in girls at T2 stage. AMH and height were negatively correlated in the girls group.

**Conclusions:**

Kisspeptin and AMH have a unique significance in the auxiliary diagnosis, the differential diagnosis, the treatment, and prognosis of sexual puberty disorder.

## 1. Introduction

Precocious puberty is traditionally defined as the onset of pubertal development before the age of eight years in girls and nine years in boys, or menstruation starts before ten years in girls in China [1]. Precocious puberty can be classified as central precocious puberty (CPP, also known as gonadotropin-dependent precocious puberty, is caused by early maturation of the hypothalamic-pituitary-gonadal (HPG) axis [2]) and peripheral precocious puberty (PPP, also known as gonadotropin-independent precocious puberty, is caused by excess secretion of sex hormones, including isolated premature thelarche (PT), or isolated androgen-mediated sexual characteristics). They all can lead to some psychological and social problems. It was found in our clinical treatment that some patients of PT developed CPP, which needs diagnosis and treatment in the early stages. The cause and auxiliary diagnosis of CPP is a focus of current research in the field [3]. Kisspeptin is now recognized as an indispensable factor in regulating puberty through FSH and LH levels [4]. Kisspeptin was found in previous studies which was higher in cases of precocious puberty [5] or not different [6]. Currently, it has not been fully elucidated how kisspeptin is involved in precocious puberty and the relationship of LH. Also involved in gonadal development is anti-Müllerian hormone (AMH). AMH is secreted by immature testicular Sertoli cells and participates in sperm production in males and is secreted by ovarian granulosa cells in women [7]. Before puberty, AMH is very low, and only after puberty, AMH secretion reached peak in females, but in males the opposite is the case. AMH has the function of regulating sex hormone production, and its level changes have been related to the onset of puberty in both sexes [8], as the different physiological structure of the gender, time, and amount of AMH secretion are different. It is not clear how AMH participates in precocious puberty.

The aim of this study was to evaluate the change of kisspeptin and AMH in children with PT or CPP or controls to provide a reference for diagnosis, treatment, and prognosis.

## 2. Methods

This study was cross-sectional. We performed a power analysis to determine the sample size, using kisspeptin as the primary outcome. With an alpha of 0.05 (one-sided test) and 80% power, a sample size of 17 was needed in the CPP group and a sample size of 33 was needed in the control group to reveal a significant difference among the groups. Assuming a dropout rate of 10%, we determined 38 newly diagnosed with CPP Chinese girls who only have breast development without menstruation and 19 Chinese boys and 19 girls in T2 newly diagnosed with PT. In addition, 25 healthy Chinese adolescent boys and 50 adolescent girls of bone age, with body mass index (BMI) similar to CPP, attending the childcare clinic in the Second Hospital of Shandong University (Jinan, China) for health examination, were recruited as healthy controls from December 2016 to August 2019. Written informed consent was provided by both parents of each child. The study was approved by the Ethics Committee of Shandong University and was carried out in accordance with the Declaration of Helsinki. The inclusion criteria for the CPP group were as follows: girls with Tanner stage 2, aged 3–8 years, meet the diagnostic criteria for CPP as at the beginning of the study [1]; they only have breast development without menstruation; and no acute diseases at least 3 months prior to the beginning of the study. The inclusion criteria for the PT group were as follows: girls with Tanner stage 2, aged 3–8 years, having leuprolide stimulation test resulting indicative of PT and boys with Tanner stage 2, aged 3–9 years, having leuprolide stimulation test resulting indicative of PT, meet the diagnostic criteria for PT [9]. All participating healthy controls, 8- to 10-year-old adolescent girls with Tanner stage 2 or 3 and 10- to 14-year-old adolescent boys with Tanner stage 2 or 3, were confirmed to be free of chronic and acute diseases, had leuprolide stimulation test, and had not used medicine for at least 3 months prior to the beginning of the study. We followed the methods of Song et al. 2018 [10]. The stage of puberty was assessed in accordance with the Tanner criteria [11]. We divided them into seven subgroups according to the Tanner subgroup and the gender subgroup: (1) the group of CPP girls; (2) the group of PT girls with Tanner stage 2; (3) the group of PT boys; (4) the group of control girls with Tanner stage 2; (5) the group of control boys with Tanner stage 2; (6) the group of control girls with Tanner stage 3; and (7) the group of boys with Tanner stage 3.

Anthropometric measurements included weight and height. The BMI was calculated from the ratio of weight/height^2^ (kg/m^2^). Body weight was measured to the nearest 0.1 kg for all subjects wearing light clothing and no shoes. Standing height was measured using a wall-mounted stadiometer in the morning to the nearest 0.1 cm.

Fasting blood samples were obtained from all recruited children after an overnight fast to measure the following parameters: follicle-stimulating hormone (FSH) and luteinizing hormone (LH) were measured by a chemiluminescence immunoassay (Immulite 2000, Siemens, Eschborn, Germany). The normal ranges in our laboratory were as follows: LH: 1.2–103 mIU/ml and FSH: 1.5–10 mIU/ml. The lowest value of LH that can be detected is 0.1 mIU/ml. The lowest value of FSH that can be detected is 0.1 mIU/ml. AMH (CSB-E12756h, purchased from Solarbio, Shanghai, PR China) and kisspeptin (CSB-EL012373HU, purchased from Solarbio, Shanghai, PR China) were detected with ELISA kit. The lowest values of AMH and kisspeptin that can be detected are 0.06 ng/ml and 0.075 ng/ml, respectively. The size of the uterus and ovary was detected by B ultrasound. The uterine volume is expressed as length multiplied by width and multiplied by height. Ovarian volume (OV) is expressed as length multiplied by width and multiplied by height and multiplied by 0.532. Bone age (BA) was performed in all children. BA was determined by radiograph of the left hand and wrist according to the method of Greulich and Pyle. MR examination of the pituitary gland excluded tumors. Leuprolide stimulation test is performed: a dose of 0.25 ug/kg triptorelin (Ferring GmbH, Germany) or a maximum dose of 0.1 mg, after injection, 30th, 60th, 90th, and 120th minutes, respectively, which measures serum FSH and LH. A cutoff value of stimulated LH ≥ 5 IU/L and a peak LH/FSH ratio >0.6 were diagnosed as puberty [12]. It is the same cutoff value for both boys and girls.

### 2.1. Statistical Analyses

Normally distributed variables are expressed as mean ± standard deviation (SD). Independent sample *t*-tests and analysis of variance were performed using SPSS software version 20.0 (SPPS, Chicago, IL, USA). Depending on the type of data distribution, Pearson's correlation analysis was used to analyze correlations in normally distributed data. Relationships among different group variables were analyzed by regression analysis. Statistical significance was defined as *P* < 0.05 for all variables.

## 3. Results

### 3.1. Baseline Characteristics

The data of 38 children with CPP (38 girls, mean age 7.6 ± 1.6 years), 38 children with PT (16 boys and 22 girls, mean age 4.9 ± 3.19 years), and 75 healthy children (36 boys and 39 girls, mean age 8.02 ± 3.83 years) were analyzed. Baseline characteristics of all subjects are described in Table 1. Renal function (urea nitrogen and creatinine) and liver function were normal. Gender, chronological age, and BA differed significantly among the three different groups. BA was significantly lower in the PT group than in the CPP group and control group. The average baseline age was 4.9 y in the PT group and 8 y in the control group, and this difference was significant; moreover, the difference remained significant when considering boys and girls separately in the PT group. No significant differences were observed in other baseline clinical data among the three groups. There is a difference between chronological age and BA in controls (*P* = 0.009). There is no difference between chronological age and BA in the PT group and CPP group. There is no T2 stage in the CPP group.

AMH decreased significantly in the CPP girls than in control girls with T2 (4.72 ± 0.98 ng/ml vs. 5.10 ± 3.64 ng/ml, *P* < 0.05), and AMH increased significantly from T2 stage to T3 stage in control girls (5.10 ± 3.64 ng/ml vs. 6.75 ± 2.29 ng/ml, *P* > 0.05). AMH decreased mildly from T2 stage to T3 stage in boys in the control group (4.91 ± 2.32 ng/ml vs. 3.41 ± 2.36 ng/ml, *P* > 0.05). Compared with the PT and control groups, AMH is the lowest in the CPP group (9.78 ± 4.77 ng/ml vs. 7.52 ± 1.52 ng/ml vs. 4.63 ± 1.18 ng/ml, *P* < 0.05). AMH was significantly higher in control girls with T2 than in control boys with T2 (5.10 ± 3.64 ng/ml vs. 4.47 ± 2.22 ng/ml, *P* < 0.05). Kisspeptin was lower in CPP girls than in the control girls with T3 (0.33 ± 0.22 ng/ml vs. 0.60 ± 0.70 ng/ml, *P* < 0.05). Kisspeptin decreased significantly in girls in the PT group (0.56 ± 0.63 ng/ml vs. 0.08 ± 0.0 ng/ml, *P* < 0.05) and increased significantly in girls in the control group from T2 stage to T3 stage (0.21 ± 0.23 ng/ml vs. 0.93 ± 0.55 ng/ml, *P* < 0.05). At T3 stage, kisspeptin was most lowest in girls in the PT group among the three groups (0.08 ± 0.0 ng/ml vs. 0.92 ± 0.55 ng/ml vs. 0.43 ± 0.16 ng/ml, *P* < 0.05). Kisspeptin did not change from T2 stage to T3 stage in boys in the control group (0.56 ± 0.85 ng/ml vs. 0.57 ± 0.40 ng/ml, *P* > 0.05). Kisspeptin was significantly higher in normal boys than in normal girls at T2 stage (0.56 ± 0.85 ng/ml vs. 0.21 ± 0.23 ng/ml, *P* < 0.05). At T3 stage, kisspeptin was slightly higher in girls than in boys (0.92 ± 0.54 ng/ml vs. 0.57 ± 0.41 ng/ml, *P* > 0.05).

**Figure 1:**
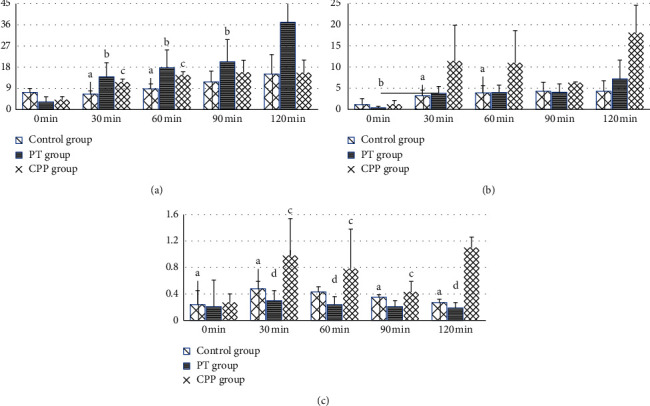
Comparison of the FSH and LH among the different groups. (a) Comparison of the FSH among the three groups. (b) Comparison of the LH among the three groups. (c) Comparison of the LH/FSH among the three groups. “A” represents comparison among the three groups, *P* < 0.05; “B” represents comparison between PT and controls, *P* < 0.05; “C” represents comparison between CPP and controls; “D” represents comparison between CPP and PT, *P* < 0.05.

FSH60 differed significantly among the three different groups. FSH30 in the CPP group was 1.7 times more than that in the control group. A similar difference was observed for FSH60. FSH30, FSH60, and FSH90 were significantly higher in the PT group than in the control group (Figure 1(a)). LH30, FSH30, and LH60 differed significantly among the three different groups. LH30 and LH60 were elevated in the CPP group compared with the PT group and control group, but no difference was observed. LH0 was significantly lower in the PT group than in the control group, and there was no difference between girls and boys in all recruited children. No difference was observed between LH ≥ 0.2 (mIU/ml) group and LH < 0.2 (mIU/ml) group except age [13]. In the girls group, LH30 in the CPP group was 3 times higher than that in the PT group (*P* = 0.007), as well as LH60 in the CPP group was 2.7 times as much as in the PT group (*P* = 0.011) (Figure 1(b)). LH30/FSH30, 60, 90, and 120 differed significantly among three different groups (Figure 1(c)).

**Figure 2:**
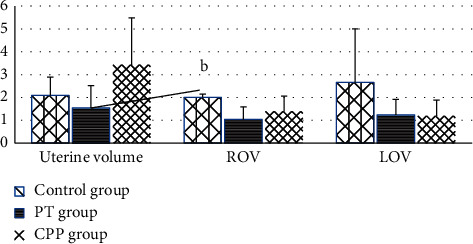
Comparison of the uterine volume and ovarian volume among the three groups. “A” represents comparison among the three groups, *P* < 0.05; “B” represents comparison between PT and controls, *P* < 0.05; “C” represents comparison between CPP and controls; “D” represents comparison between CPP and PT, *P* < 0.05.

**Figure 3:**
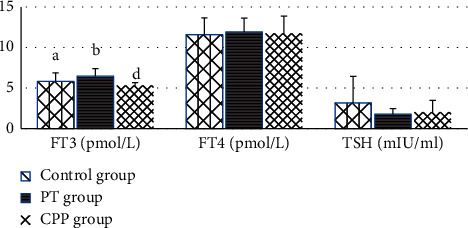
Comparison of thyroid hormone among the three groups. Values are expressed as the mean ± SD. “A” represents comparison among the three groups, *P* < 0.05; “B” represents comparison between PT and controls, *P* < 0.05; “C” represents comparison between CPP and controls; “D” represents comparison between CPP and PT, *P* < 0.05.

A significant increase in right OV (ROV) was observed in the control group, compared with the PT group (2.00 ± 0.16 ml vs. 1.04 ± 0.56 ml) (Figure 2). FT3 was markedly elevated in the PT group compared with the CPP group and control group (Figure 3). Progesterone in the control group was 35% of those for the PT group.

### 3.2. Correlation

AMH and progesterone were positively correlated (Pearson's *ρ* = 0.51; *P* = 0.001), and FSH0 and prolactin were also positively correlated (Pearson's *ρ* = 0.366; *P* = 0.025). ROV showed a negative correlation with FSH30, FSH60, and FSH90 (Pearson's *ρ* = −0.659, *P* = 0.014; *ρ* = −0.623, *P* = 0.022; *ρ* = −0.619, *P* = 0.042). The left OV (LOV) was negatively correlated with FSH30, FSH60, and FSH90 (Pearson's *ρ* = −0.711, *P* ≤ 0.001; *ρ* =−0.668, *P* ≤ 0.001; *ρ* =−0.765, *P* ≤ 0.001). Testosterone and LH were positively correlated (Pearson's *ρ* = 0.556, *P* ≤ 0.001). Kisspeptin showed a positive correlation with testosterone and LH0 (Pearson's *ρ* = 0.69, *P* = 0.009; *ρ* = 0.80, *P* = 0.001), and testosterone and LH0 were also positively correlated in the boys group (Pearson's *ρ* = 0.87, *P* ≤ 0.001). The AMH showed a positive correlation with progesterone and FSH120 in the girls group (Pearson's *ρ* = 0.58, *P* = 0.003; *ρ* =0.97, *P* = 0.006). AMH and height were negatively correlated in the girls group (Pearson's *ρ* = −0.63, *P* = 0.008). ROV showed a negative correlation with FSH30 and FSH60 (Pearson's *ρ* = −0.59, *P* = 0.04; *ρ* = −0.58, *P* = 0.004) in the girls group. LOV showed a negative correlation with FSH30, FSH60, and FSH90 (Pearson's *ρ* = −0.69, *P* = 0.013; *ρ* = −0.64, *P* = 0.024; *ρ* = −0.74, *P* = 0.014) in the girls group. FSH0 and prolactin were also positively correlated (Pearson's *ρ* = 0.42; *P* = 0.04) in the girls group. LH and testosterone were also positively correlated (Pearson's *ρ* = 0.66, *P* ≤ 0.001) in the girls group.

## 4. Discussion

The progression rate of PT can hardly be predicted at the point of diagnosis. Any decision for treatment initiation relies on the continued monitoring of pubertal progression, which is currently the most valuable tool available for clinicians. Basal FSH and LH concentrations have limited diagnostic utility in distinguishing children with CPP from those with benign pubertal variants [2]. GnRH is the initiating and core factor in the HPG axis, which is influenced by many neurotransmitters and neuropeptides, liking kisspeptin and AMH [14]. In this study, we investigated how kisspeptin and AMH levels change during normal and abnormal sexual development.

CPP is pathologic in up to 75 percent of cases in boys [15]. Therefore, only girls were recruited for the CPP group in our study. We found that there were no differences in kisspeptin among the three groups. As a result, it was discussed according to the Tanner stage. At T2 stage, no difference in kisspeptin was observed between the PT group and the control group, but at T3 stage, kisspeptin was significantly lower in the PT group compared with the CPP and control groups, which could be used as a method of distinguishing CPP and PT. It can be used as an indicator of whether PT will convert to CPP. Kisspeptin in the CPP group and control group did not differ at T3 stage. Thus, kisspeptin may not reflect the difference between CPP children's and normal children's future fertility function. From T2 to T3 stage, the girls of kisspeptin increased significantly in CPP and controls, but decreased in PT. However, the boys rarely changed. Kisspeptin may not play a very important role in male sexual development. We believe that kisspeptin can be a follow-up indicator of the PT group. If kisspeptin decreases with the increase of the Tanner stage, PT may not convert into CPP. For girls, it is a good follow-up indicator of PT, but for boys, it is not. This also reflects that kisspeptin plays an important role in puberty development, perhaps not be a trigger marker. Therefore, kisspeptin offers promising biomarkers in predicting pubertal progression.

There was no significant difference in AMH among the three groups. Therefore, it was discussed according to the Tanner stage. At T2 stage, AMH was similar in the PT group and the control group and also similar in boys and girls. AMH may not be a good marker of early diagnosis in PT or CPP. At T3 stage, AMH in girls was significantly higher than that in boys, same as the previous study [16, 17]. However, we found that AMH is the lowest in CPP girls at T3 stage. Results indicate that AMH can be a means of identifying PT and CPP. AMH is a marker of the primordial follicle pool [8] and might reflect alterations in the rate of ovarian follicle recruitment and follicle activity, respectively [18]. Therefore, AMH can reflect the ovarian function which is not well developed in CPP. It helps us predict the future fertility of CPP girls. We found that AMH decreased significantly in boys, but increased significantly in girls from T2 to T3 stage. Meanwhile, AMH was a positive correlation with progesterone and FSH. Previous studies have also found that FSH has been demonstrated to directly stimulate AMH production [19]. AMH in CPP boys is lower at diagnosis and AMH can be back to normal after treatment, accompanied by an increase in testosterone [20]. Therefore, we confirm that AMH can be used as a sign of improvement of CPP in boys. AMH was negatively correlated with height in the girls group which includes PT, CPP, and controls, same as the previous study [21]. This will help us to decide whether we should add growth hormones to improve the final height in the treatment of CPP. The ovary volumes of CPP are larger than the same age of the control group and are smaller than the same bone age of the control group, indicating that the development is not good enough. AMH and FSH were positively correlated. FSH was also positively correlated with the ovary volumes. AMH reflects ovarian function. Overall, these data support the conclusion that AMH is helpful for the diagnosis and treatment of precocious puberty in children.

## 5. Conclusions

Kisspeptin and AMH are important sexual development factors. They may not be good indicators for early diagnosis. However, they have a unique significance in the auxiliary diagnosis, the differential diagnosis, the treatment, and prognosis of sexual puberty disorder.

## Figures and Tables

**Table 1:** General characteristics of the three groups.

	Control group (*n* = 75)	PT group (*n* = 38)	CPP group (*n* = 38)
Age (years)	8.02 ± 3.83	4.9 ± 3.19	7.8 ± 1.6
Boys (*n*)	36	16	0
Girls (*n*)	39	22	38
Height (cm)	120.3 ± 19.95	107.7 ± 23.1	132.0 ± 11.5
BMI (kg/m^2^)	16.1 ± 2.8	15.94 ± 1.71	18.8 ± 2.8
Bone age (years)	8.69 ± 3.30	4.65 ± 2.94	9.66 ± 1.15
Kisspeptin (ng/ml)	0.50 ± 0.69	0.44 ± 0.58	0.42 ± 0.16
Progesterone (ng/ml)	0.64 ± 0.41	1.77 ± 4.91	0.28 ± 0.20
AMH (ng/ml)	4.98 ± 2.75	5.64 ± 4.11	4.63 ± 1.18
Prolactin (ng/ml)	12.55 ± 9.23	8.22 ± 3.34	9.18 ± 4.40
Estradiol (pg/ml)	18.09 ± 18.55	17.75 ± 15.16	23.84 ± 24.38
Testosterone (ng/ml)	0.28 ± 0.81	0.02 ± 0.04	0.01 ± 0.00
ROV (ml)	2.00 ± 0.16	1.04 ± 0.56	1.39 ± 0.68
LOV (ml)	2.66 ± 2.34	1.25 ± 0.68	1.19 ± 0.70

## Data Availability

The datasets generated and/or analyzed during the current study are available from the corresponding author on reasonable request.
